# Measuring Geographic Atrophy Area Using Column-Based Machine Learning Software on Spectral-Domain Optical Coherence Tomography versus Fundus Auto Fluorescence

**DOI:** 10.3390/bioengineering11080849

**Published:** 2024-08-19

**Authors:** Or Shmueli, Adi Szeskin, Ilan Benhamou, Leo Joskowicz, Yahel Shwartz, Jaime Levy

**Affiliations:** 1 Department of Ophthalmology, Hadassah-Hebrew University Medical Center, Ein-Karem, Jerusalem 91120, Israel; orchuk86@gmail.com (O.S.); yahels@hadassah.org.il (Y.S.); 2 School of Computer Science and Engineering, The Hebrew University of Jerusalem, Givat-Ram, Jerusalem 9190401, Israel; adi.szeskin@mail.huji.ac.il (A.S.); ilan.benhamou@mail.huji.ac.il (I.B.); josko@cs.huji.ac.il (L.J.)

**Keywords:** optical coherence tomography, autofluorescence, measurement, geographic atrophy

## Abstract

Background: The purpose of this study was to compare geographic atrophy (GA) area semi-automatic measurement using fundus autofluorescence (FAF) versus optical coherence tomography (OCT) annotation with the cRORA (complete retinal pigment epithelium and outer retinal atrophy) criteria. Methods: GA findings on FAF and OCT were semi-automatically annotated at a single time point in 36 pairs of FAF and OCT scans obtained from 36 eyes in 24 patients with dry age-related macular degeneration (AMD). The GA area, focality, perimeter, circularity, minimum and maximum Feret diameter, and minimum distance from the center were compared between FAF and OCT annotations. Results: The total GA area measured on OCT was 4.74 ± 3.80 mm^2^. In contrast, the total GA measured on FAF was 13.47 ± 8.64 mm^2^ (*p* < 0.0001), with a mean difference of 8.72 ± 6.35 mm^2^. Multivariate regression analysis revealed a significant correlation between the difference in area between OCT and FAF and the total baseline lesion perimeter and maximal lesion diameter measured on OCT (adjusted *r*^2^: 0.52; *p* < 0.0001) and the total baseline lesion area measured on FAF (adjusted *r*^2^: 0.83; *p* < 0.0001). Conclusions: We report that the GA area measured on FAF differs significantly from the GA area measured on OCT. Further research is warranted in order to determine the clinical relevance of these findings.

## 1. Background

Geographic atrophy (GA) is a common cause of severe vision loss due to age-related macular degeneration (AMD). As of October 2023, Pegcetacoplan and Avacincaptad pegol have been approved as new treatment options for GA [1]. Given the severe vision loss that GA can cause, it is of utmost importance to detect and monitor GA at its early stages. This underscores the significance of our research in providing a better understanding of GA measurement using FAF and OCT, which could potentially improve early detection and monitoring strategies.

GA can be detected and measured using color fundus photography (CFP), fundus autofluorescence (FAF), infrared (IR) imaging, and spectral-domain optical coherence tomography (SD-OCT, hereafter referred to as OCT). GA has been recently classified as having increasing levels of severity. cRORA (complete retinal pigment epithelium (RPE) and outer retinal atrophy) is defined as the complete loss of photoreceptors and RPE, thus providing a specific anatomical endpoint that may serve as a valuable marker for future GA clinical trials [2]. Importantly, cRORA can be detected on OCT but cannot be identified on FAF or CFP [2]. Moreover, most studies that measure GA use either CFP or FAF. Second, GA areas that show hypo-autofluorescence on FAF also show correlated retinal sensitivity loss [3,4,5]. Finally, FAF has been approved by the US FDA as a clinical endpoint for use in GA trials [3,6,7].

On the other hand, OCT is more available than FAF nowadays and does not cause the light aversion seen with the bright light used for FAF or CFP [2,4]. Moreover, OCT is advantageous compared to FAF in foveal assessment due to natural foveal hypo-autofluorescence caused by the masking effect of the yellow macular pigment [8]. Finally, specific retinal layer damage, as well as cRORA, can be detected on OCT but cannot be detected on FAF or CFP [2].

Two recent studies measured GA area using FAF and OCT with cRORA criteria and found no significant difference between OCT and FAF with respect to total baseline GA area [9,10].

In the past decade, automatic algorithms for GA annotation using OCT showed good correlations with human expert manual annotations [11,12,13,14,15]. Likewise, the automatic annotation of GA using FAF is also in use, e.g., the Region Finder software (Ver. 2.6.6.0, Heidelberg Engineering Inc., Heidelberg, Germany) [16].

Recently, we developed a custom software platform for machine learning-based automatic image analysis that can be used to annotate GA in OCT scans using cRORA criteria [17]. Unlike FAF, OCT B-scans can provide anatomical staging of GA at a given time point [2]. Here, our goal was to measure and compare using semi-automated annotation of measure and compare GA area annotated using OCT with GA area annotated using FAF and then attempt to identify potential shape descriptors of GA on OCT and FAF that are correlated with the difference in GA area measured using OCT and FAF.

## 2. Methods

### 2.1. Study Design, Patient Selection, and Data Collection

In this retrospective study, we collected data for eyes with advanced dry AMD in patients examined between 2012 and 2021 at the Department of Ophthalmology, Hadassah Medical Center, Jerusalem, Israel. This study was conducted in strict accordance with the ethical guidelines outlined in the Declaration of Helsinki and received approval from the Institutional Review Board (IRB). Due to the retrospective nature of the study, the Institutional Review Board of Hadassah Medical Organization waived the need for obtaining informed consent (approval number HMO-382-19).

The study population included patients over 55 years of age who presented with GA secondary to AMD, with no signs of current or prior choroidal neovascularization on OCT and with suitable quality FAF and OCT scans. Patients had to have OCT scans with at least 61 B-scan slices per cube scan and a high signal-to-noise ratio, as evaluated qualitatively by the study graders (O.S., Y.S.). Eyes were excluded from the study when retinal atrophy was due to high myopia (defined as a refractive error greater than −6 diopters) and/or macular dystrophy. Patients with occluded ocular media, e.g., significant cataract, causing low image quality, were excluded. A total of 36 eyes in 24 patients were included.

### 2.2. FAF Image Acquisition

FAF images were obtained with the Spectralis HRA-OCT system (Heidelberg Engineering GmbH, Heidelberg, Germany) using an excitation wavelength of 488 nm and an emission wavelength of 500–700 nm, with a field of view of 30° × 30°. The fovea served as the center of the scan, and the resolution was 768 × 768 pixels.

### 2.3. FAF Image Analysis and Semi-Automated Annotation

FAF images were exported from the Heidelberg Eye Explorer software in Tagged Image File Format (TIFF) format and uploaded to ImageJ 1.53, as described previously [10]. The images were semi-automatically annotated in ImageJ software by two experienced masked graders (O.S., Y.S.), as previously described [18,19,20].

All 36 images were cropped to include the macula with the entire GA area. The scale of each image was then standardized using the accompanying 200-micron scale that appears in the images.

To mark the lesion area, the graders used the ImageJ “wand” tool, which creates automatic selection by tracing either objects of uniform color or thresholded objects. Using the “brightness/contrast improvement” function in ImageJ, the graders then increased the contrast between the GA area and the surrounding non-GA area. The users then manually adjusted the “wand” tool threshold until the highlighted area matched the area of the GA lesion. Both the threshold and the noise were adjusted to exclude extraneous vessels and the peripapillary area.

Atrophic lesions annotated on FAF were defined as being at least 250 microns in diameter and appeared as hypoautofluorescent with a black level similar to the optic nerve. Some GA lesions can contain slightly gray (rather than black) areas in some locations due to the presence of residual hyper-reflective material in the bed of atrophy [10].

In each annotated FAF scan, the lesion contour was highlighted using a specific color for easy detection by thresholding. The annotated scans were loaded into an integrated development environment (PyCharm Ver. 2 January 2020, JetBrains) using Python Library Scikit-Image. Next, for a given annotated FAF scan, a binary mask of the lesion’s contours was extracted using a thresholding technique. The measurement scale located at the bottom-left of the image was also extracted. The contour mask was then filled to produce a region segmentation mask of the GA lesions; specifically, pixels that were inside the annotated lesion contour were labeled as lesion pixels, while the remaining pixels were labeled as background pixels. The resulting binary image (called a segmentation mask) contained multiple regions (called connected components) representing the lesion. Next, each connected component in the segmentation mask was assigned a label. Then, the following properties of the labeled image regions were computed: the GA area, focality, perimeter, circularity, minimum and maximum Feret diameter, and minimum distance from the center. Finally, the results were converted to the appropriate unit of measurement using the extracted scale.

In addition, Region Finder software (Heidelberg Engineering Inc.) annotations were made by two graders (O.S., Y.S.) to validate the GA areas annotated in FAF with ImageJ. The annotation in Region Finder was added in a semi-automated fashion, as described previously [16].

### 2.4. OCT Image Acquisition

OCT images were acquired using the Spectralis OCT system. The imaging protocol included up to 97 B-scan slices per cube scan, with a width and height of 20°; specifically, 25, 2, and 9 eyes had 61, 73, and 97 B-scans per cube scan, respectively. The technical properties of the IR images and OCT images and the computational methods used are described elsewhere [21].

### 2.5. OCT Image Analysis and Semi-Automated Annotation

We utilized artificial intelligence tools to create a method for automatically detecting and measuring cRORA and other AMD atrophies in OCT scans and visualizing them in corresponding infrared images. The technique analyzes light scattering patterns in vertical pixel-wide vectors within OCT slices containing atrophy using a specialized column-based convolutional neural network (CNN). Each column is classified based on a 3D column patch comprising neighboring columns. Atrophy columns are then projected onto the IR image to identify and segment atrophy lesions, measuring their areas. Through previous experiments on 106 OCT scans (consisting of 5207 slices) with 2952 atrophy segments and 1046 atrophy lesions related to cRORA, we achieved a mean F1 score of 0.78 (with a standard deviation of 0.06) and an AUC of 0.937, indicating results close to observer variability [17].

The criteria used for GA annotation in OCT scans were based on the cRORA definition from the “Consensus Definition for Atrophy Associated with Age-Related Macular Degeneration on OCT” (CAM) [2]. Lesions of cRORA were annotated on each B-scan when all of the following criteria were present: (1) choroidal hyper-transmission, (2) RPE attenuation or disruption, (3) overlying photoreceptor degeneration, (4) a minimum diameter of 250 microns, and (5) an absence of scrolled RPE or other signs of an RPE tear.

Two experienced graders (O.S., Y.S.) semi-automatically annotated GA lesions in 36 OCT cube scans. Our custom software (herein referred to as “OCT cRORA Editor”) annotated GA in the OCT B-scans semi-automatically. This OCT deep learning platform has been previously tested in another study by our team and showed good agreement with expert retina physicians [17].

The graders used our image analysis software to automatically annotate the GA lesion columns in OCT B-scans and then manually add corrections. The annotations were then projected onto the corresponding IR image and the lesion area and shape descriptive features, e.g., the focality, circularity, perimeter, diameter, and distance of the lesion from the center, as described elsewhere [22].

The over-annotation of lesions not related to AMD was prevented by ignoring peripapillary atrophic lesions not connected to macular GA lesions (further details provided in a previous study) [22].

To compare the OCT annotations with the FAF annotations, we performed a point-based manual registration between each FAF scan and the corresponding IR image. Due to differences between OCT and FAF with respect to image acquisition, we did not use an automated registration algorithm. For each scan, we chose three geographical points on the FAF image and the three corresponding points on the IR image. Next, the differences in scaling, rotation, and translation (affine registration) between the two images were computed and used to register the FAF image onto the IR image space. The registration was also used to localize the fovea centralis on the FAF image.

### 2.6. Measured Parameters

Inter-grader agreement was tested by calculating the Dice coefficient [23] for both OCT and FAF annotations. In OCT and FAF scans with high inter-grader agreement (Dice coefficient > 90%), the outcome measure was calculated as the average of both graders’ measurements. When the Dice coefficient was lower than 90%, the annotations were adjudicated by a senior grader annotation (JL). We measured GA area and shape-descriptive parameters using both the OCT and FAF scans; the majority of these parameters were assessed previously in the context of GA using FAF [24,25], CFP [26], or—less frequently—OCT [27].

As reported previously for FAF scans [25], we also measured the following four additional baseline shape-descriptive factors: the minimum and maximum lesion diameters (Feret_min_ and Feret_max_, respectively); the focality index, defined as the number of lesions with an area >0.05 mm^2^; and the circularity index, defined as [4π × (area/perimeter^2^)]. In addition, we measured the total baseline lesion perimeter [28], as well as the minimum distance between the lesion and the fovea centralis.

### 2.7. Study Outcomes

The primary outcomes (GA area, focality, perimeter, circularity, minimum Feret diameter, maximum Feret diameter, and minimum distance from the center) were compared between the OCT and FAF scans. In addition, the putative correlation between the primary outcomes on OCT and the difference in GA area between OCT and FAF were analyzed using univariate and multivariate regression analyses.

### 2.8. Statistical Analysis

The study sample did not satisfy the assumption of normal distribution as quantified with the Shapiro–Wilk test. Comparisons of the primary outcomes between OCT and FAF were performed using the Wilcoxon test. Unless indicated otherwise, summary data are listed as the mean ± SD and range. In addition, univariate regression analysis was used to test the potential effects of OCT and FAF descriptive factors on the total difference in annotated GA area between OCT and FAF; any factors that had a significant impact on the difference in GA area between OCT and FAF in the univariate analysis were then included in a multivariate stepwise linear regression analysis. The multicollinearity of independent variables was ruled out using a variance inflation factor of <2.5 for all variables.

### 2.9. Subgroup Analysis

The relatively low scanning density of 61 B-scans per cube scan in some eyes may have had a negative impact on the results due to the insufficient imaging of the total area of GA. Therefore, we also performed a subgroup analysis of the nine eyes with 97 B-scans.

## 3. Results

A total of 36 pairs of OCT cube scans and FAF scans obtained for 36 eyes in 24 patients with dry AMD were included in our analysis. The inter-grader agreement with respect to GA annotation in the OCT and FAF scans was measured using the 36 individual FAF images and OCT scans from the study. The resulting Dice coefficient was 0.86 ± 0.09 for the OCT annotations and 0.96 ± 0.03 for the FAF annotations, indicating a good degree and excellent degree, respectively, of inter-grader agreement. The Dice coefficient for the FAF annotations with Region Finder was 0.95 ± 0.04.

### 3.1. GA Outcome Measures

**Figure 1:**
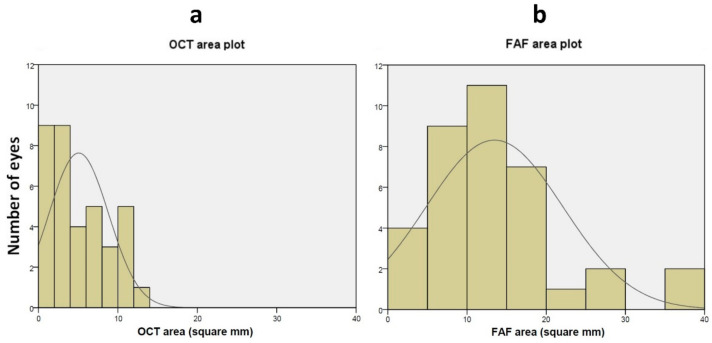
Distribution of GA area annotated using OCT (**a**) and FAF (**b**). (**a**) Distribution of the number of eyes plotted against GA area (in mm^2^) measured using OCT. The solid line represents a Gaussian fit of the data. (**b**) Distribution of the number of eyes plotted against GA area (in mm^2^) measured using FAF. The solid line represents a Gaussian fit of the data. GA = Geographic atrophy; OCT = Optical coherence tomography; FAF = Fundus auto-fluorescence.

**Figure 2:**
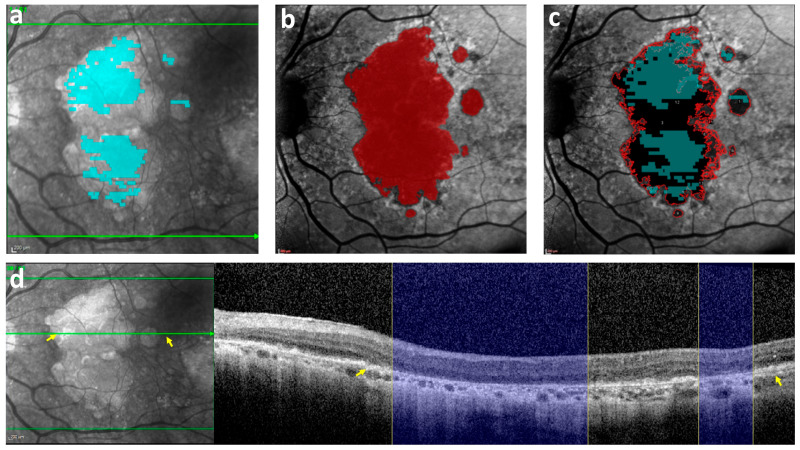
An index case illustrating the difference in GA area between OCT and FAF. (**a**) The GA annotation on an OCT B-scan using the cRORA criteria (in cyan) was projected on the corresponding IR image, showing a measured GA area of 11 mm^2^. (**b**) The GA annotation on FAF (in red), with a measured GA area of 26 mm^2^. (**c**) Both the OCT annotation from (**a**) (cyan) and the FAF annotation from (**b**) (red outline) were projected on the FAF image. (**d**) The B-scan on the right corresponds to the hyper-reflective area on the green line in the IR image on the left (the areas between the two yellow arrows in the IR image and B-scan match). Right: The cRORA evident in the OCT B-scan (indicated by the blue columns) corresponds only partially to the hypo-autofluorescent areas seen in the level of the B-scan section on FAF in (**c**). GA = Geographic atrophy; OCT = Optical coherence tomography; FAF = Fundus auto-fluorescence; cRORA = Complete retinal pigment epithelium and outer retinal atrophy.

Table 1 summarizes the GA outcomes measured for the paired OCT and FAF scans for all 36 eyes included in our analysis. The total GA area measured with FAF was significantly larger than the total area measured with OCT (see also Figure 1 and Figure 2). The total GA measured in FAF with Region Finder was smaller than that measured with ImageJ but larger than that measured on OCT (Supplementary Table S1).

Similarly, all other shape descriptive factors were measured larger on FAF as compared with those on OCT (Table 1).

### 3.2. Correlation between the OCT Shape Descriptors and the Difference in GA Area Measured between FAF and OCT

Table 2 summarizes the correlation between the OCT shape descriptors and the difference in GA area measured between FAF and OCT. Univariate regression analysis revealed a significant correlation between the FAF-OCT GA area difference and the following variables: total lesion area, perimeter, focality, and maximum lesion Feret. These four variables were then included in a multivariate analysis using stepwise linear regression. The multivariate model had an adjusted *r*^2^ value of 0.52, and the total lesion perimeter and maximum lesion Feret were significantly associated with the difference in GA area between FAF and OCT (Table 2).

### 3.3. Correlation between the FAF Shape Descriptors and the Difference in GA Area Measured between FAF and OCT

Table 3 summarizes the correlation between the FAF shape descriptors and the difference in GA area measured between FAF and OCT. Univariate regression analysis revealed a significant correlation between the FAF-OCT GA area difference and the following variables: total lesion area, perimeter, minimum distance from center, and maximum lesion Feret. These four variables were then included in a multivariate analysis using stepwise linear regression. The multivariate model had an adjusted *r*^2^ value of 0.83, and only the total lesion area was significantly associated with the difference in GA area between FAF and OCT (Table 3).

### 3.4. Subgroup Analysis

The GA outcomes measured for the paired OCT and FAF scans in a subgroup of nine eyes with OCT scans containing 97 slices are summarized in Table 4. This subgroup analysis revealed significant differences in total lesion area, focality, circularity, and maximum lesion Feret.

We also examined the putative correlation between the OCT shape descriptors and the difference in GA area measured between FAF and OCT for this subgroup (Supplementary Table S2). The resulting multivariate model had an adjusted *r*^2^ value of 0.68, with focality in OCT being the only variable significantly associated with the difference in GA area measured between FAF and OCT.

## 4. Discussion

We measured GA area semi-automated annotation in 36 eyes with dry AMD using FAF images and compared the results with the semi-automated annotation of GA area measured on OCT using CAM criteria [2]. We found a significant difference in GA area between these two imaging modalities. Moreover, the difference in GA area between FAF and OCT was correlated with the total baseline lesion perimeter and maximal diameter on OCT and the total baseline lesion area on FAF, and our subgroup multivariate analysis revealed a correlation between focality on OCT and the difference in GA area between FAF and OCT.

Although most previously published studies used FAF to detect and measure GA [3,28], the signal on FAF can be affected by media opacity and optical aberrations. In addition, comparing absolute FAF signal intensity both between patients and in an individual patient over time is challenging [5]. In contrast, measuring GA based on the OCT-specific cRORA criteria may provide more accurate results, as the measurements reflect specific anatomical changes rather than signal intensity. Thus, we sought to examine the difference in GA area between OCT using cRORA criteria and FAF.

In contrast with previously reported results, we found that the total GA area was significantly larger when measured using FAF compared to that measured using OCT. Several factors may explain this apparent discrepancy. First, we performed OCT annotations using the more specific and less sensitive endpoint of atrophy, i.e., cRORA, compared to the more general forms of retinal atrophy measured with FAF. Second, previously reported evidence of RPE dysmorphia surrounding GA on histology of donor retinas [29] may contribute to this difference, as RPE dysmorphia can allow light to penetrate the RPE, manifesting as hypo-autofluorescence on FAF [30]. In contrast, RPE dysmorphia is not necessarily associated with all of the CAM criteria for cRORA on OCT B-scans. Third, a well-known issue with FAF is the normal gradual decrease in the autofluorescence signal toward the center of the macula due to a masking effect of the yellow macular pigment [8]. This issue often results in a tendency to over-annotate GA in FAF in the foveolar area and may partially contribute to the difference in GA area between OCT using cRORA criteria and FAF.

Cleland et al. measured GA area in 70 eyes using FAF and OCT with cRORA criteria, finding no significant difference [9]. Similarly, Velaga et al. measured GA area in 270 eyes in 172 patients using FAF and OCT with cRORA criteria and found no significant difference in total baseline GA area between OCT and FAF [7]. It is worth mentioning that Velaga et al. used a lower minimum cut-off for GA lesion annotation (125 microns) for cRORA annotation, which may have increased the measured area of annotated GA in their OCT scans.

In our study, the GA area difference between OCT and FAF was correlated with the lesion perimeter on OCT in multivariate analysis and on FAF in univariate analysis. Also, the GA area difference between OCT and FAF was correlated with multifocality on OCT. Although no previous studies found a correlation between GA shape descriptors and the difference in GA area between OCT and FAF, some studies found a correlation between both GA lesion perimeter and focality and a more rapidly growing GA lesion. In a recent meta-analysis, Shen et al. imaged 3489 eyes with GA using CFP, FAF, and/or OCT and demonstrated an association between multifocality and GA area progression [28]. The authors concluded that the faster rate of GA area growth was related to the proportional increase in total lesion perimeter, which is correlated with a higher focality index [28]. The same group documented 318 eyes with GA using CFP and found that the rate of GA area progression was strongly associated with the baseline lesion perimeter; however, the GA perimeter-adjusted growth rate was not correlated with baseline focality, circularity, or total lesion size [31]. Recently, our team examined 33 eyes with GA and found that baseline GA lesion focality was correlated with the rate of GA area progression on multivariate analysis; interestingly, however, the lesion’s perimeter was associated with the rate of GA area progression only on univariate analysis [22].

Our current findings and the previously reported results suggest that a larger baseline lesion perimeter is correlated with a higher rate of GA growth. Larger numbers of healthy RPE cells exposed to the advancing border of retinal atrophy may promote cellular events that drive apoptosis and/or immune-related cell death.

Our analysis revealed a correlation between the lesion’s distance from the center in FAF and the difference in GA area between FAF and OCT on univariate analysis. However, an increased rate of GA progression was previously reported in patients with extra-foveal lesions [32], which would likely be associated with the difference in GA area between FAF and OCT in our study.

Maximum and minimum lesion Feret were previously reported as being correlated with the rate of GA progression [25]. However, in our study, only maximum lesion Feret was correlated with the difference in GA area between FAF and OCT, and only on univariate and multivariate analysis.

In their previous study, Shen et al. examined 237 eyes imaged using CFP and found that the baseline GA area was correlated with the rate of GA lesion growth [33]. In the AREDS-2 study, which followed a cohort of 517 eyes imaged using CFP, the baseline GA area was directly correlated with the rate of GA lesion growth in medium-sized GA lesions, with extremely small and extremely large GA lesions showing relatively slow growth [34]. Notably, in our study, the difference in GA area measured between FAF and OCT was correlated with the baseline GA area on OCT in the univariate analysis and with the baseline GA area on FAF in the multivariate analysis, with a much higher correlation coefficient for the GA area on FAF. This may be explained by the variable timing of capturing GA lesions. More mature lesions may have grown to their final area captured on both OCT and FAF, while growing lesions may show a nearly final area only on FAF, as explained previously by RPE dysmorphia being more visible on FAF and the more exhaustive cRORA definition on OCT.

Together, these findings indicate that lesions with a larger perimeter, multifocality, and a larger minimal distance from the center are more likely to be undergoing growth. It is therefore likely that this growth will be identified first on FAF but only later identified as cRORA, due to the more exhaustive criteria required to meet the definition of cRORA and the above-mentioned RPE dysmorphia that surrounds GA lesions and can contribute to GA appearing as hypo-autofluorescence on FAF before complete atrophy becomes apparent as cRORA on OCT. This earlier appearance on FAF may account for the difference between OCT and FAF with respect to the GA area and may be associated with the aforementioned GA lesion perimeter on OCT. This correlation between the GA perimeter on OCT and the difference in the GA area between FAF and OCT may have implications for studying new GA therapies, as FAF is currently the only FDA-approved modality for measuring GA endpoints in clinical trials [7,35].

Our study has several limitations. First, we included a relatively small patient size in our analysis. Second, the software that we developed for annotating GA in the OCT scans has not been independently validated. Nevertheless, Cleland et al. used the custom-developed OCT Split Tool (EyeKor, Inc., Madison, Wisconsin) for their OCT annotations [9]. Lastly, the relatively low scanning density (with 61 B-scans per cube scan in some cases) may have negatively affected the results due to the insufficient imaging of the total GA area and/or a failure to identify small multifocal lesions; however, the difference in total GA area between OCT and FAF remained significant in our subgroup analysis of 9 eyes with 97 slices per OCT cube scan.

On the other hand, our work has several strengths. First, the inter-grader agreement of OCT annotations in our study was high, while previous reports stress that grading OCT scans for cRORA in a consistent, repeatable, and reproducible manner is challenging [36,37]. Recently, a total of 60 eyes were imaged using OCT, and cRORA was annotated by twelve formally trained ophthalmologists; inter-reader agreement based on Gwet’s first-order agreement coefficient (AC_1_) was substantial for cRORA (AC_1_ = 0.68) [36]. In a separate study by Chandra et al., a total of 50 eyes were imaged by OCT, and cRORA was annotated by five retina-trained ophthalmologists, with significant variability in the diagnosis of cRORA on SD-OCT, with Cohen’s kappa values ranging from 0.28 to 0.92 [37]. Of note, the FAF inter-reader agreement in our work was very high, with a DICE of 0.96. Second, although our study includes OCT scans with a lower B-scan density than the scans reported by Cleland et al. and Velaga et al., we consider this a strength of our study, as it reflects real-world OCT scans, which typically have a low scan density. Finally, we quantitatively analyzed the putative correlations between GA shape descriptors on OCT and FAF and the difference in GA area between OCT and FAF using a multivariate analysis, revealing several significant correlations.

## 5. Conclusions

We found that the lesion area measured with FAF was significantly higher than the area measured with OCT. In addition, the lesion perimeter and maximal diameter on OCT and the baseline area on FAF were associated with the difference in GA area between OCT and FAF. Together, these results may help researchers estimate the potential difference in the lesion area when annotating GA using the relatively new OCT B-scan method compared to the more traditional FAF scan method. Prospective studies with larger cohorts are warranted in order to determine the effect of OCT B-scan density, the OCT device, and/or the cut-off point for cRORA annotation on the difference in GA area annotation between OCT and other imaging modalities such as FAF. Such information will likely have high clinical relevance, particularly given that increasing the accuracy of GA measurements will help determine the eligibility of patients with GA for inclusion in clinical trials.

## Figures and Tables

**Table 1:** GA outcome measured using OCT and FAF.

*p*-Value	FAF	OCT	Factor
	(*n* = 36 Eyes)	(*n* = 36 Eyes)	
**<0.0001**	13.47 ± 8.64	4.74 ± 3.80	Total lesion area (mm^2^)
	(0.24; 37.98)	(0.15; 13.23)	
**0.002**	42.36 ± 22.31	33.38 ± 19.74	Perimeter (mm)
	(2.93; 97.17)	(1.63; 77.44)	
**0.001**	4.54 ± 3.37	8.53 ± 6.17	^1^ Focality
	(1.00; 15.00)	(1.00; 31.00)	
**<0.0001**	0.34 ± 0.18	0.49 ± 0.14	^2^ Circularity
	(0.10; 1.15)	(0.07; 0.75)	
**<0.0001**	1.43 ± 1.04	0.31 ± 0.39	Minimum distance from center (mm)
	(0; 3.62)	(0; 1.89)	
**0.002**	1.18 ± 1.62	0.31 ± 0.71	Minimum lesion Feret (mm)
	(0.03; 5.22)	(0.05; 3.55)	
**<0.0001**	4.70 ± 1.59	2.81 ± 1.48	Maximum lesion Feret (mm)
	(0.74; 8.14)	(0.52; 6.77)	

Data are presented as the mean ± standard deviation and range. *p*-values <0.05 are presented in bold. GA = Geographic atrophy; OCT = Optical coherence tomography; FAF = Fundus auto-fluorescence. ^1^ Focality index was defined as the number of lesions with an area >0.05 mm^2^. ^2^ Circularity index was defined as 4π × (area/perimeter^2^).

**Table 2:** Correlation between OCT shape descriptors and FAF-OCT area difference (*n* = 36 eyes).

Univariate Regression Analysis		
Variable	^1^ r	*p*-Value
Total lesion area (mm^2^)	0.408	**0.007**
Perimeter (mm)	0.68	**<0.0001**
^2^ Focality	0.58	**<0.0001**
^3^ Circularity	0.05	0.391
Minimum distance from center (mm)	0.12	0.250
Minimum lesion Feret (mm)	−0.22	0.102
Maximum lesion Feret (mm)	0.35	**0.018**
**Multivariate Regression Analysis**		
**Variable**	**Estimated β**	***p*-Value**
Total lesion area (mm^2^)	−0.15	0.66
Perimeter (mm)	0.31	**<0.0001**
Focality	0.07	0.77
Maximum lesion Feret (mm)	−1.63	**0.047**
Adjusted *r*^2^	**0.52**	

*p*-values < 0.05 and the adjusted r^2^ are presented in bold. OCT = Optical coherence tomography; FAF = Fundus auto-fluorescence. ^1^ Pearson’s correlation coefficient. ^2^ Focality index was defined as the number of lesions with an area >0.05 mm^2^. ^3^ Circularity index was defined as 4π × (area/perimeter^2^).

**Table 3:** Correlation between FAF shape descriptors and FAF-OCT area difference (*n* = 36 eyes).

Univariate Regression Analysis		
Variable	^1^ r	*p*-Value
Total lesion area (mm^2^)	0.92	**<0.0001**
Perimeter (mm)	0.74	**<0.0001**
^2^ Focality	0.32	0.09
^3^ Circularity	−0.12	0.25
Minimum distance from center (mm)	−0.44	**0.004**
Minimum lesion Feret (mm)	0.11	0.48
Maximum lesion Feret (mm)	0.87	**<0.0001**
**Multivariate Regression Analysis**		
**Variable**	**Estimated β**	***p*-Value**
Total lesion area (mm^2^)	0.67	**<0.0001**
Perimeter (mm)	0.31	0.34
Minimum distance from center (mm)	0.07	0.13
Maximum lesion Feret (mm)	−1.63	0.62
Adjusted *r*^2^	**0.83**	

*p*-values < 0.05 and the adjusted r^2^ are presented in bold. OCT = Optical coherence tomography; FAF = Fundus auto-fluorescence. ^1^ Pearson’s correlation coefficient. ^2^ Focality index was defined as number of lesions with an area >0.05 mm^2^. ^3^ Circularity index was defined as 4π × (area/perimeter^2^).

**Table 4:** GA outcomes measured using OCT (with 97 slices per cube scan) and FAF.

*p*-Value	FAF	OCT	Factor
	(*n* = 9 Eyes)	(*n* = 9 Eyes)	
**0.001**	9.85 ± 5.08	3.35 ± 3.18	Total lesion area (mm^2^)
	(2.65; 16.73)	(0.57; 10.38)	
0.51	42.36 ± 22.31	37.13 ± 24.33	Perimeter (mm)
	(16.16; 52.58)	(8.68; 84.93)	
**0.01**	4.11 ± 2.93	13.00 ± 7.92	^1^ Focality
	(1.00; 8.50)	(5.00; 26.00)	
**0.04**	0.36 ± 0.15	0.49 ± 0.08	^2^ Circularity
	(0.12; 0.67)	(0.38; 0.59)	
0.08	1.20 ± 1.09	0.40 ± 0.30	Minimum distance from center (mm)
	(0.11; 3.62)	(0; 0.99)	
0.06	1.35 ± 1.78	0.08 ± 0.03	Minimum lesion Feret (mm)
	(0.03; 4.62)	(0.05; 0.14)	
**<0.0001**	3.97 ± 1.00	2.23 ± 1.10	Maximum lesion Feret (mm)
	(2.03; 5.17)	(0.80; 4.23)	

Data are presented as the mean ± standard deviation and range. *p*-values < 0.05 are presented in bold. GA = Geographic atrophy; OCT = Optical coherence tomography; FAF = Fundus auto-fluorescence. ^1^ Focality index was defined as number of lesions with an area >0.05 mm^2^. ^2^ Circularity index was defined as 4π × (area/perimeter^2^).

## Data Availability

The corresponding author has full access to all the data in the study and takes responsibility for the integrity of the data and the accuracy of the data analysis as well as the decision to submit for publication. All data are available from the corresponding author on request.

## Supplementary Materials

The following supporting information can be downloaded at: https://www.mdpi.com/article/10.3390/bioengineering11080849/s1, Table S1: Total GA semi-automatically annotated using FAF with Region Finder Vs. FAF with ImageJ Vs. OCT with the “Editor” Software; Table S2: Correlation between OCT (with 97 slices per cube scan) shape descriptors and FAF-OCT area difference (*n* = 9 eyes).
