# Supplementary material for: Measuring Geographic Atrophy Area Using Column-Based Machine Learning Software on Spectral-Domain Optical Coherence Tomography versus Fundus Auto Fluorescence
Source: Bioengineering (Basel). 2024 Aug 19;11(8):849. doi: 10.3390/bioengineering11080849 (PMC11351153; doi:10.3390/bioengineering11080849)
Supplement: Supplementary file 1 [file bioengineering-11-00849-s001.zip › Table S2.pdf]

Table S2. GA outcomes measured using OCT (with 97 slices per cube scan) and FAF.

Factor	OCT (n=9 eyes)	FAF (n=9 eyes)	P-value
Total lesion area (mm^2)	3.35±3.18 (0.57; 10.38)	9.85±5.08 (2.65; 16.73)	**0.001**
Perimeter (mm)	37.13±24.33 (8.68; 84.93)	42.36±22.31 (16.16; 52.58)	0.51
[1]Focality	13.00±7.92 (5.00; 26.00)	4.11±2.93 (1.00; 8.50)	**0.01**
[2]Circularity	0.49±0.08 (0.38; 0.59)	0.36±0.15 (0.12; 0.67)	**0.04**
Minimum distance from center (mm)	0.40±0.30 (0; 0.99)	1.20±1.09 (0.11; 3.62)	0.08
Minimum lesion Feret (mm)	0.08±0.03 (0.05; 0.14)	1.35±1.78 (0.03; 4.62)	0.06
Maximum lesion Feret (mm)	2.23±1.10 (0.80; 4.23)	3.97±1.00 (2.03; 5.17)	**<0.0001**

Data are presented as the mean ± standard deviation and range. P-values <0.05 are presented in bold. GA=Geographic atrophy; OCT=Optical coherence tomography; FAF=Fundus auto-fluorescence. [1]Focality index was defined as number of lesions with an area >0.05 mm^2. [2]Circularity index was defined as 4π x (area/perimeter^2).
